# The global failure of facing the pandemic

**DOI:** 10.1080/16549716.2022.2124645

**Published:** 2022-10-26

**Authors:** Luis Eugenio Portela Fernandes de Souza, Marcia Caldas Castro, Eduardo Hage Carmo, Maurício Polidoro

**Affiliations:** a Institute of Saúde Coletiva, Federal University of Bahia, Salvador, Brazil; b Department of Global Health and Population, Harvard School of Public Health, Boston, MA, USA; c Environmental Sciences Area, Federal Institute of Rio Grande do Sul, Porto Alegre, Brazil

**Keywords:** Global health, Covid-19, pandemic

## Abstract

The COVID-19 pandemic outbreak in late 2019 has had social, political, and economic consequences worldwide. However, its emergence was not a surprise. In 2015, a Panel organised by the World Health Organization highlighted the importance of learning about the crisis caused by the Ebola epidemic. In 1992, the Committee on Emerging Microbial Threats to Health of the US Institute of Medicine warned of the possibility of an emerging global microbial threat. In this text, we point out five arguments that reveal the global failure in facing the pandemic: (1) deficiency in the global alert system and the fragility of the International Health Regulations (IHR-2005), (2) problems of the international response to the pandemic, related to global health governance, (3) the dispersed global adoption of the elimination strategy (zero Covid) widely seen as a policy of restriction of freedom instead as a strategy of inequities reduction, (4) fragile control of the disease with a narrow reading of the associated problems, and (5) global setbacks in achieving the Sustainable Development Goals in the context of ongoing neoliberal national policies. Finally, we argue that overcoming the weaknesses discussed requires strengthening health systems in all their components and expanding social welfare policies.

**Figure:**
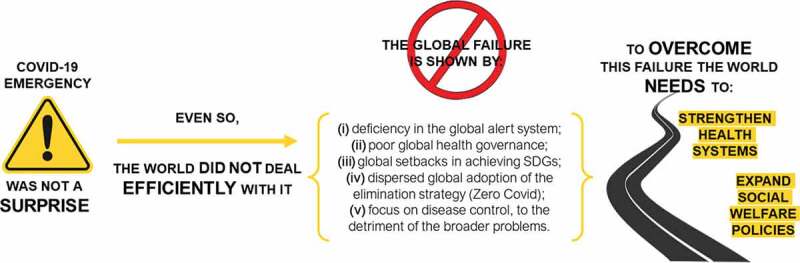

## Background

The infection of humans with a novel coronavirus in late 2019 and the subsequent pandemic were by no means a surprise. At least since 2015, the United Nations predicted the possibility of this occurrence based on the previous epidemics of the first coronavirus, different strains of influenza virus (H5N1, H1N1), and Ebola [1].

Long before that, in 1992, the Committee on Emerging Microbial Threats to Health of the US Institute of Medicine mentioned this risk related to factors such as (a) demography and human behaviour, (b) technology and industry, (c) economic development and land use, (d) travel and international trade, (e) adaptation and microbial change, and (f) collapse of public health measures [2].

And yet, despite studies and warnings, the emergence of SRAS-CoV-2 in Wuhan has found countries and the entire United Nations system unprepared. Then, how have the multilateral system and countries dealt with the Covid-19 emergency?

## Five arguments for the global failure of facing the pandemic

To begin with, it should be stressed that the World Health Organization (WHO) strictly followed the International Health Regulations (IHR-2005) guidelines. It was based on IHR-2005 that the WHO declared, on 30 January 2020, coronavirus an international public health emergency, the highest alert level predicted. This declaration, however, was not enough to trigger vigorous preparedness and response actions in most countries. Thus, in an attempt to sound louder the alert, the WHO declared, on 11 March, that a ‘pandemic’ was underway, a situation not foreseen in the IHR-2005.

Referring to these initial moments, the Independent Panel on Pandemic Preparedness and Response concluded, in May 2021, ‘that the legally binding IHR is a conservative instrument (…), it serves to constrain, rather than facilitate, quick action and that the precautionary principle was not applied (…) when it should have been’ [3].

Criticising the Independent Panel’s findings, the Review Committee on the Functioning of the International Health Regulations (2005) during the COVID-19 response presented a report to the 2021 World Health Assembly, stating that ‘the IHR are not deficient, but their implementation by member states and by WHO was inadequate’ [4].

Regardless of whether the fragility results from the text or the implementation of the IHR-2005, both reports point to deficiencies in the global alert and response mechanisms. More important, however, is recognising that IHR-2005 is not the only weak point in the global fight against the Covid-19 pandemic.

Therefore, it is necessary to recognise several weaknesses in the international response to the pandemic. In a report presented to the 2021 World Health Assembly, the Independent Oversight and Advisory Committee for the WHO Health Emergencies Program, established in 2016, pointed out the main difficulties in dealing with Covid-19: insufficient surveillance actions, monitoring and risk management, including early detection, tracking, and case isolation; absence or inadequacy of risk communication; lack of a global genomic surveillance network; deficiencies in national health systems in terms of care for the infected and the sick, including the protection of health workers; and inability to ensure adequate provision of equipment and supplies, especially equitable access to vaccines [5].

It is important to note that weaknesses in the international response to the pandemic refer to the health governance system as a whole [6,7] and not specifically to the WHO leadership and staff. There is no doubt that WHO leaders and professionals have acted intensely and even courageously, providing timely and consistent technical guidance, perhaps with a single severe flaw regarding the delay in admitting the transmission of the coronavirus was airborne [8].

It must be admitted that the causes of these difficulties are not new but rather well known. Within the scope of the multilateral system, the support that the WHO usually gives to different countries – especially low-income countries – has been compromised by under-financing and dependence on donations that generally have a destination defined by the donor. This reduction in financial transfers to the WHO by the Member States has caused a shortage of health personnel, whether in central bodies or the national representations. Added to this are questioning the guidelines or even attacks on the WHO by political leaders from powerful countries and the increase in geopolitical tensions.

One can observe performance inferior to that of governance in global health in other instances of the United Nations system. Regarding the fight against Covid-19, the United Nations General Assembly, the Security Council, and the Economic and Social Council remained at the rhetorical level with few actions effectively carried out. It is worth adding the ineffectiveness of the World Trade Organization, which, given the boycott of the wealthiest countries, has not even discussed a simple proposition of temporary suspension of intellectual property rights related to products and technologies aimed at combating the current health emergency for two years.

The causes of the failure of facing the pandemic are not only at the global level but also associated with a lack of investment and staff shortages in national health systems. If disinvestment and scarcity are chronic problems in low-income countries, they have become serious problems in several high-income countries due to privatisation and state reduction measures adopted in the last three or four decades.

Not all nations, however, have disinvested in their social protection systems. And those that strengthened their social protection systems, including health services, fared better in the face of the health emergency and its socio-economic consequences, as a comprehensive study shows, involving 28 countries [9]. Thus, where there were comprehensive responses to adapt health services, preserve the functions and resources of social protection systems and reduce situations of social vulnerability, the results of the fight against Covid-19 were better, both in health and social terms.

Schematically, comparing countries, one can identify two primary strategies for coping with the pandemic: elimination and mitigation. The first is characterised by taking the necessary actions to interrupt community transmission, while mitigation refers to the adoption of measures to reduce the number of cases in order not to overload health systems.

**Figure 1.**
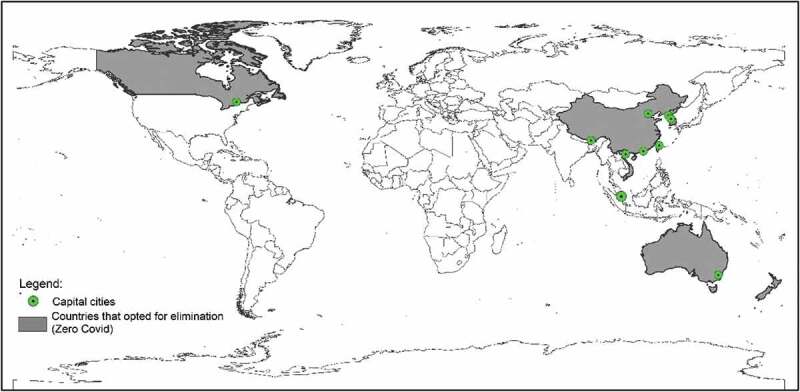
Countries that opted for elimination.

Several reviews [10–13], bringing together studies that assess the performance of countries, show that those who opted for elimination performed better. Deaths from Covid-19 per million inhabitants in countries that opted for elimination (see Figure 1) (China, Australia, Japan, New Zealand, South Korea, and others) were lower than in countries that preferred mitigation. Elimination also outperformed mitigation regarding average GDP growth over most of the analysed periods.

The coloured countries on the map represent countries that have adopted the zero Covid policy. Source: [10, 11, 12, 13].

Certain critics of the elimination strategy claim that it leads to the restriction of individual freedoms. However, considering measures such as the closing of schools, shops and restaurants and restrictions on the movement of people, the studies show that freedoms were more severely affected in countries that opted for mitigation, given that restrictive measures were more frequent.

In short, the elimination strategy – also called Covid Zero – produced better results both in terms of health and maintenance of economic activities, even before the availability of vaccines.

At the end of 2020, the start of vaccination raised the hope of controlling the pandemic. The epidemiological situation has improved in countries with high coverage rates, significantly reducing the number of severe cases and deaths.

However, the inequity of access to vaccines between countries prevents any control of the pandemic in the short term. As of 13 April 2022, while high-income countries had already vaccinated 71.9% of their population, low-income countries had only vaccinated 15.5% with at least one dose [14]. It should be noted that the leading cause of this iniquity is the political choice of the corporations and governments of rich countries to protect and promote the profit of some companies, even at the cost of the lives and health of billions of people around the world.

In addition to prolonging the suffering of the poorest, low vaccine coverage contributes to the emergence of new variants and subvariants, which have caused new waves of cases, hospitalisations, and deaths, showing that vaccination alone cannot control pandemics.

The analysis of the global confrontation of the pandemic so far demonstrates that the control of the Covid-19 is fragile and may lead to a picture of endemicity interspersed with outbreaks. How the world fights Covid-19 contributes to setbacks in achieving the Sustainable Development Goals. More than four years of progress against poverty has been erased by Covid-19, which has hampered global economic recovery and caused the first rise in between-country income inequality in a generation [15].

## Implications

Our analysis makes it clear that overcoming the health crisis would require tackling the structural causes of the pandemic. Indeed, transformations in the mode of social production would be necessary to enable the distribution of wealth consistent with the contributions and needs of each one, as well as the widespread adoption of preservationist and conservationist practices (reduction of deforestation, expansion of natural parks, strengthening of agroecology and organic sustainable extractivism, etc.).

In the health sector, specifically, it would be a matter of developing primary prevention actions, including the surveillance of wild animals and the regulation of the bushmeat trade, in line with the One Health approach.

## Limitations

This study brings a broad reflection on the performance of the multilateral system and all countries facing the pandemic. Although based on publicly available data and scientific research results, this study is not a neutral reflection but is guided by the ethical principle of equity in health.

## Conclusion

In sum, the world was unprepared and has faced the Covid-19 pandemic badly, despite the efforts of the WHO and the dedication of millions of health professionals in all countries of the world. In managing the pandemic, most nations favoured the less effective mitigation strategy, and no government took action to tackle the deep causes of the health and social crisis.

So far, no interventions have been aimed at modifying the current development model as part of the strategies to combat Covid-19. This lack of interventions on the structural causes of the multiple crises stems from the political strength of minorities who benefit from the current model, disproportionately appropriating socially generated wealth. They are the major shareholders of large companies that dominate the global market in all sectors of the economy and dictate the formulation and implementation of policies in each country and in multilateral forums, where they sometimes disguise themselves as philanthropists.

## Paper context

An increasingly voluminous scientific production has been dedicated to the topic of the Covid-19 pandemic. However, there is still a need to broaden the understanding of the mistakes made in facing the pandemic at the global level. Aware of this, researchers from the International Relations Committee of the Brazilian Association of Collective Health in Brazil (ABRASCO) point out in this text, in five arguments, the mistakes made and indicate the possibilities of changes and preparation for the next pandemic.
